# Impact of Fatigue on the Skin: Pilot Study on “996” Work‐Life Pattern in China and the Influence of Their Lifestyle on the Skin

**DOI:** 10.1111/jocd.70679

**Published:** 2026-01-21

**Authors:** Xiaoyu Ma, Li Ma, Yimei Tan, Wencai Jiang, Pujos Muriel, Florante Ricarte, Doucet Olivier

**Affiliations:** ^1^ Coty, Research and Development Shanghai China; ^2^ Department of Skin and Cosmetic Research Shanghai Skin Disease Hospital Shanghai China; ^3^ COTY, Research and Development Monaco Monaco; ^4^ COTY Research and Development Morris Plains New Jersey USA

**Keywords:** fatigue, fine lines, lifestyle impact, skin aging, skin elasticity

## Abstract

**Background:**

Modern living encouraged longer working hours and an increased sense of fatigue. In China, a rising trend called “996”, meaning working from 9 a.m. to 9 p.m., 6 days per week in succession, has raised concerns about the impact of fatigue on health, specifically on skin aging.

**Methods:**

A one‐week, single center, self‐controlled clinical study was conducted to evaluate the impact of fatigue on skin phenotypes, through the enrollment of 31 Chinese women with a 996 work‐life pattern based on Life Style questionnaires and Chalder fatigue scale. Clinical grading and instrumental measurements were conducted before the working week, after 1, 3, and 5 working days.

**Results:**

The fatigue score of the participants accumulated during the working week. From clinical grading, periocular fine lines, fine lines of the cheeks, and forehead wrinkles were significantly worsened in the working week compared to the weekend. Skin elasticity parameters R0, R5, and R7 were also significantly worse compared to the weekend. Significantly increased porphyrin, yellowness, and decrease of redness of skin during working times were also observed. Additionally, the clinical evaluations were well recorded with self‐perceptions from the questionnaire.

**Conclusion:**

Fatigue is an inescapable stressor in modern life that impacts skin's health and appearance by accelerating signs of aging, altering skin color, and increasing risk of acne. This is a pilot study analyzing the population with intensive work‐life patterns, serving as a reference to comprehensively assess the impact of modern living on skin.

## 1 Introduction

Modern living has narrowed the boundary between work and life. Long working hours encourage highly intensive work‐life patterns. According to a World Health Organization (WHO) and International Labour Organization (ILO) report, as early as in 2016, 488 million people globally have already been exposed to long working hours [1]. In China, individuals also suffer from intensive work‐life imbalance, which has developed into a rising trend called “996”, meaning working from 9 a.m. to 9 p.m. for 6 days per week in succession. Such phenomena have raised great concerns about the impact of fatigue on one's state of health.

As defined by the Canadian Centre for Occupational Health and Safety (CCOHS), fatigue is the state of feeling very tired, weary, or sleepy, resulting from insufficient sleep, prolonged mental or physical work, or extended periods of stress or anxiety [2]. Therefore, fatigue cannot be recognized as pure psychological stress or lack of sleep alone. Rather, the state of fatigue is of great complexity and can result from a combination of behavioral, psychological, or dietary perturbations.

Fatigue could trigger a series of health issues [3], encompassing cognitive, mental, metabolic, cardiovascular, gastrointestinal, and neurological disorders. Additionally, fatigue may also cause harm to the skin. As reported by Skoie [4], fatigue is deeply connected with the innate immune system and cellular stress responses, which accompany inflammation and oxidative stress. In inflammatory skin diseases such as atopic dermatitis or psoriasis, fatigue not only serves as a symptom, but also as an aggravating factor [4, 5].

However, apart from the influence on diseased skin, little is known about the impact of fatigue on normal skin. Previous studies have shown how psychological stress and sleep deprivation can modulate skin phenotype. For example, the “brain‐skin axis” was established as transmitting psychological stress to skin through neuronal signaling [6] to induce skin barrier malfunction, chronic inflammation, and skin aging [7, 8]. In other studies, it was found that inadequate sleep time disturbs the skin circadian rhythm [9, 10], thus contributing to oxidative stress, DNA damage, barrier function [10] and skin aging [11]. Though these studies have revealed the potential impact of fatigue, none have shown a direct link between fatigue and skin conditions, since fatigue is a more complicated and comprehensive state than psychological stress or sleep deprivation. Based on these findings, Flament et al. investigated the impact of working on the skin phenotype among Caucasians and Asians [12, 13]. It was found that a single day's work was sufficient to induce a change in facial aging signs. However, how fatigue influences individuals under extremely intensive work‐life patterns during the work cycle remains unknown.

**FIGURE 1:**
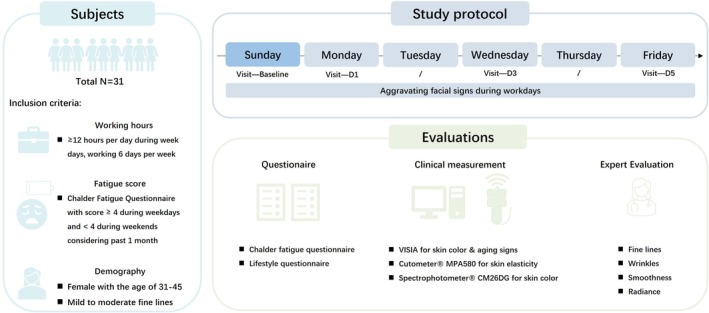
Illustration of the subject characteristics, study protocol and evaluation parameters of the clinical trial.

Here, we conducted a one‐week, single center, self‐controlled clinical study (Figure 1) to investigate the impact of fatigue on Asian skin. By enrolling subjects with 996 work‐life patterns and high fatigue status, we were able to track the fatigue sensation and skin phenotype change throughout the working week via questionnaires, clinical grading, and instrumental measurements.

## 2 Materials and Methods

### 2.1 Participants

A total of 33 Chinese female participants aged 30–45 years old and with mild to moderate fine lines were enrolled into the study, and 31 out of 33 completed the whole study. All participants were required to finish a lifestyle questionnaire and Chalder fatigue questionnaire [14] during screening (Tables S1 and S2). The participants had a highly intensive work‐life pattern, namely working over 12 h per day for 6 days in a week in succession, and only relaxing on Sunday. The score of Chalder fatigue questionnaire ≥ 4 during the weekdays and < 4 on the rest day were required. The candidates who met the inclusion criteria were recruited to take part in the formal study.

### 2.2 Clinical Design

After enrolling in the formal study, each participant had a clinical evaluation and instrumental measurement on Sunday. The participants were instructed to use only basic moisturizing cream during the testing period. Then, participants were given verbal and written instructions regarding study requirements/restrictions and a Daily Usage Diary to record the use of the products each day.

Participants were required to return to the testing facility on Monday, Wednesday, and Friday evening. For these visits, the participants were asked not to apply sunscreen or makeup. During each visit, participants were instructed to wash their whole face first and acclimate for 30 min under controlled temperature (18°C–22°C) and humidity (40%–60%) to allow consistency. After performing the Chalder fatigue questionnaire, they underwent clinical evaluations and device measurement.

### 2.3 Skin Measurement

Clinical evaluation was performed by a board‐certified dermatologist at each visit, and measurements included dullness/radiance, roughness/smoothness, firmness, pores, forehead wrinkles, glabellar wrinkles, nasolabial fold wrinkles, periocular fine lines, fine lines on the cheek, and jawline contour, using a 0–9 point scale (smallest increment of 0.5 points).

For device measurements, VISIA CR (Canfield Scientific Inc. USA) was employed to capture redness and spots on the cheek, wrinkles on the forehead and eye area, and porphyrin under UV light. Cutometer dual MPA 580 (Courage&Khazaka, Germany) was applied to determine the elastic properties of the epidermis, including major parameters R0, R2, R5, and R7. Spectrophotometer CM26DG (Konica Minolta, Japan) was used to measure skin color and glossiness.

### 2.4 Statistical Analysis

All data were analyzed using the SPSS statistical software 22.0 for Windows. For qualitative variables, descriptive analysis was presented as Median (Min, Max). For quantitative variables, descriptive analysis was presented as mean ± SEM. Analysis of the normality distribution of the data was evaluated by means of one sample Shapiro–Wilk test. According to data distribution, data sets were analyzed using one of the two following methods: Paired Student *t‐*test and Wilcoxon Signed Rank test. For clinical evaluation, Wilcoxon Signed Rank test was used for each parameter. All statistical tests were 2‐sided at significant level with *p* < 0.05.

## 3 Results

### 3.1 Participant Demography

Based on the inclusion criteria, 33 participants were enrolled in the study, with 2 participants lost during the follow‐ups due to personal reasons. The participants' ages ranged from 31 to 45 years old, with an average of 37.5 ± 4.8 years old. The skin types of the participants were collected via questionnaire, and over 74.2% of the participants had oily to combination and dry to combination skin type. Meanwhile, 61.29% identified their skin as slightly sensitive. The demographic characteristics of all participants are detailed in Table 1.

**TABLE 1:** Demographic characteristics of all participants.

**Demography**	
Age (years)	37.5 ± 4.8 (mean ± SD)
**Skin type**	
Oily	3 (9.68%)
Dry	5 (16.13%)
Oily to combination	13 (41.94%)
Dry to combination	10 (32.26%)
Normal	0 (0%)
**Skin sensibility**	
Not sensitive	8 (25.81%)
Slight sensitive skin	19 (61.29%)
Moderate sensitive skin	4 (12.90%)
Severe sensitive skin	0 (0%)
Not sure/Don't know	0 (0%)

### 3.2 Fatigue Sensation

**FIGURE 2:**
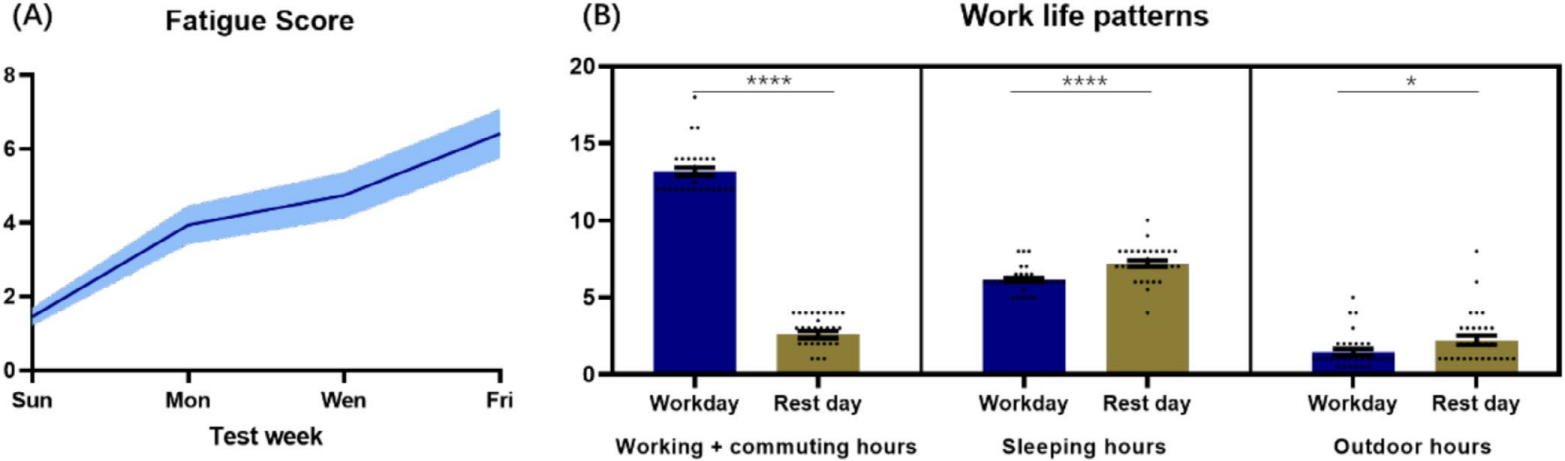
The (A) fatigue sensation and (B) work‐life patterns during the workdays and rest day (Sunday). *, **** significantly different between workday and rest day *p < 0.05 and ****p < 0.0001.

During the testing period, results showed that the fatigue score of the participants was 1.45 ± 0.24 on Sunday evening and reached 3.94 ± 0.52, 4.74 ± 0.62, and 6.42 ± 0.67 respectively on Monday, Wednesday, and Friday, showing an increasing trend during the work week (Figure 2A). Additionally, the average working hours (including commuting hours) of subjects during workdays reached 13.16 ± 0.26 h and reduced to 2.60 ± 0.23 h during the rest day. The sleeping and outdoor time during workdays were 6.17 ± 0.25 and 1.45 ± 0.21, respectively, which increased to 7.25 ± 0.30 and 2.23 ± 0.30 during the rest day (Figure 2B). The prolonged working hours and shortened sleeping and outdoor time were well accorded with the fatigue score.

### 3.3 Impact of Fatigue on Health and Skin Through Self‐Perception

As shown in Table 2, the top 5 health issues associated with fatigue were sleep, mental health, physical health, dietary issues, and social issues. For sleep issues, the most frequently encountered problems were irregular sleep patterns, light sleep, and difficulty falling asleep. For mental health issues, the top 3 threats were loss of memory, anxiety, and irascibility. As for physical health issues, 89.66% of the participants considered skin issues the highest concern, followed by hair loss and endocrine disorder. For dietary issues, longing for heavily flavored foods, over‐eating, and no appetite arose as the top concerns. In addition, some participants also complained about the lack of friends or family companionship, which hampered their social behaviors.

**TABLE 2:** Self‐perception of fatigue related health issues.

Self‐perception of fatigue related health issues^a^		
	Participant selected (*n*)	Percentage (%)
Sleep issue	31	100
(I) Irregular sleep	23	74.19
(II) Difficult to fall asleep	17	54.84
(III) Dreaminess	15	48.39
(IV) Light sleep	19	61.29
(V) Insomnia	10	32.26
(VI) Else	0	0
Dietary issue	20	64.52
(I) No appetite	10	50.00
(II) Overeating	10	50.00
(III) Unbalanced nutrition	9	45.00
(IV) Prefer high calories	9	45.00
(V) Heavy flavor	13	65.00
Physical issue	29	93.55
(I) Hair loss	22	75.86
(II) Obesity	15	51.72
(III) Skin issue	26	89.66
(IV) Endocrine disorders	22	75.86
(V) High blood pressure, lipid and blood sugar	7	24.14
(VI) Immunity	19	65.12
(VII) Gastrointestinal discomfort	8	27.59
Mental issue	30	96.77
(I) Loss of memory	27	90.00
(II) Anxiety	23	76.67
(III) Irascibility	20	66.67
(IV) Low spirit	16	53.33
(V) Instable emotion	15	50.00
Social issue	15	48.39
(I) Lack friend reunion	10	66.67
(II) Lack family companionship	12	80.00
(III) Else: no social time, no free time	2	13.33

^a^ For each category, subjects were allowed to choose no more than 5 answers.

**FIGURE 3:**
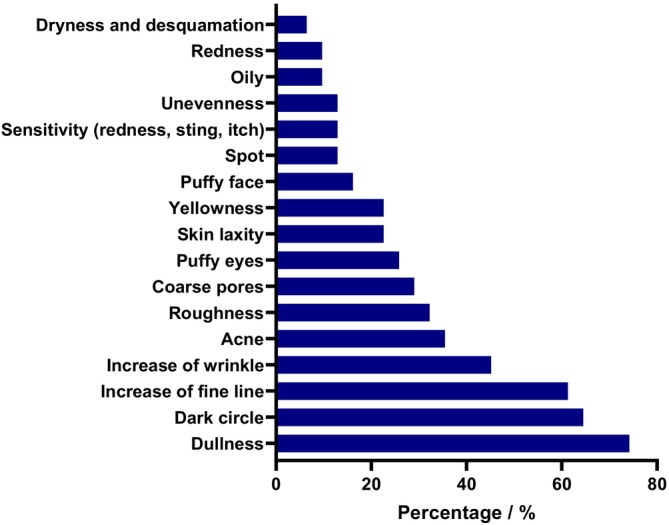
The major skin issues caused by intensive work‐life patterns from the perspective of participants.

Among different skin issues, the participants agreed that dullness was their most concerning problem, followed by dark circles and increased fine lines. Apart from the top 3 problems mentioned, about 1/3 of the subjects also considered increased wrinkles, acne, and skin roughness as the major perceived skin issues (Figure 3).

### 3.4 Impact of Fatigue on Skin Through Clinical Measurement

**FIGURE 4:**
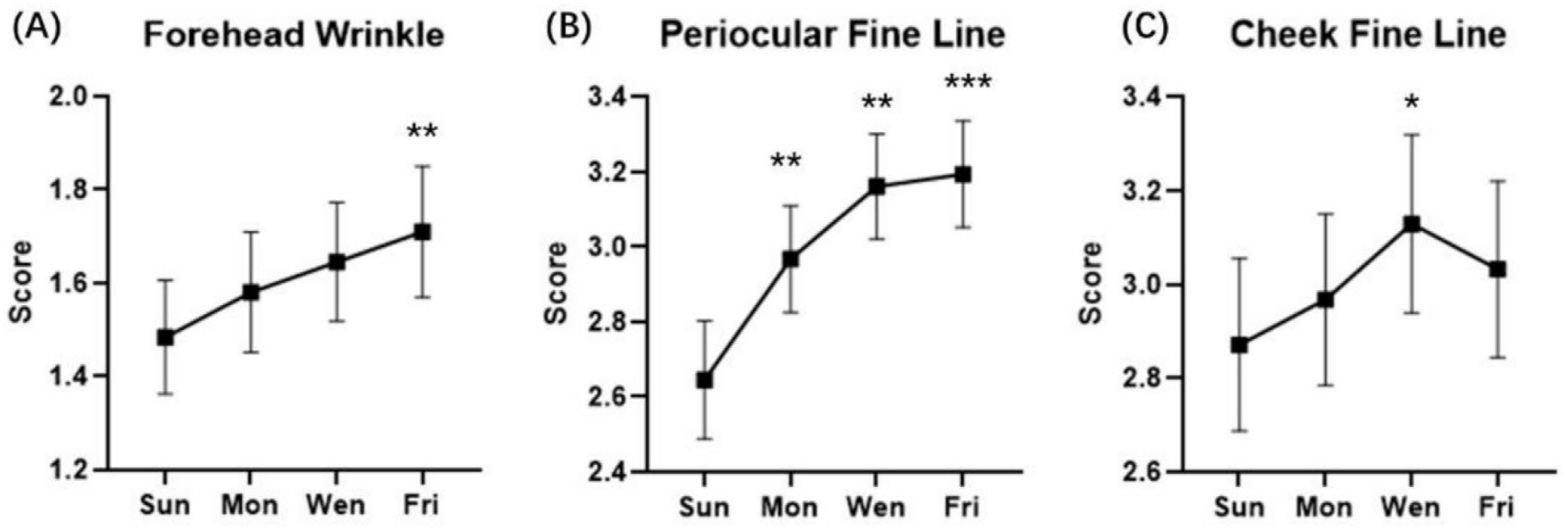
Clinical evaluation of (A) forehead wrinkle, (B) periocular fine line, and (C) cheek fine line *, **, *** significantly different from Sunday baseline results (*p* < 0.05, *p* < 0.01 and *p* < 0.001).

Based on clinical grading results (Figure 4), periocular fine lines significantly increased on Monday, Wednesday, and Friday, compared with Sunday as baseline. The fine lines of the cheeks also worsened during weekdays, but a significant difference was only observed on Wednesday. The wrinkle score gradually rose during weekdays and peaked on Friday.

**FIGURE 5:**
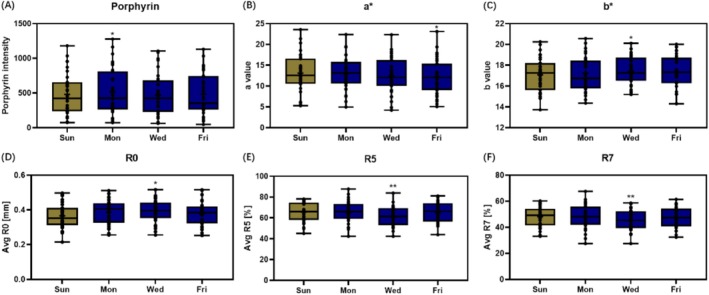
VISIA CR measurements of (A) porphyrin and (B) a, Spectrophotometer measurements of (C) b, and Cutometer measurements of (D) R0, (E) R5 and (F) R7 on participants of intensive work‐life patterns during the workdays and rest day. *, ** significantly different from Sunday baseline results (*p* < 0.05 and *p* < 0.01).

Through instrument measurements, porphyrin, skin color, and elasticity alterations were observed during weekdays as shown in Figure 5. The porphyrin increased significantly on Monday and decreased on Wednesday and Friday, but with no significant difference. The a* value exhibited a descending trend during the test period and significantly decreased on Friday. The b* value increased during weekdays and was significantly higher than baseline on Wednesday, indicating skin yellowness. As measured by Cutometer, both R5 and R7 were significantly worse on Wednesday compared to the baseline, revealing that the elasticity of skin was hampered during workdays. Instead, R0 peaked on Wednesday, which showed a significant difference compared to that at baseline.

## 4 Discussion

Mental or physical exhaustion caused by fatigue under a highly intensive work‐life pattern has increasingly drawn public attention due to its detrimental effects on both performance and health. Fatigue usually arises from inadequate sleep and disruption of the normal circadian rhythm, accompanied by less outdoor activities and change of dietary habits, thus leading to psychological stress, physical weariness, and even emotional instability [15]. In the dermatologic field, fatigue has been studied previously in disease models, but few studies focus on the direct impact of fatigue on normal skin. This study is the first to investigate the effects of fatigue on a highly intensive work‐life pattern group's skin phenotype throughout an entire workday‐rest day cycle. The study confirmed that fatigue from intensive work‐life pattern was sufficient to induce a negative change in skin phenotype in 1 week.

From the results of questionnaires, the average working hours of enrolled subjects reached 13.16 h, nearly 80 h per week, surpassing the standard working hours (35–40 h/week) [16] and even the threshold of the long‐working‐hour classification (≥ 55 h) [16]. Meanwhile, as indicated by the Fatigue Scale, the fatigue level was the lowest on Sunday (rest day), and gradually increased during the weekdays, suggesting the self‐perceived fatigue was cumulative. As previously reported in a real‐time investigation on nurses working 12‐h shifts [17], fatigue increased over the work period and was greater when working overnight, which aligned with the fatigue increase observed in our study. It is worth noting that the participants enrolled in the study should meet the fatigue threshold and “996” work‐life pattern during the past 1 month, so it is thus speculated that the possible alteration of skin phenotypes might also be the result of cumulative tiredness.

Inferred from the Life Style Questionnaire, the self‐perceived health issues encompassed physical, mental, sleep, dietary, and even social dimensions, which further verified the complex impact of fatigue. It was worth noting that the DASS‐21 results (Figure S1) further showed that nearly 96.8% participants experienced at least one negative psychological status including stress, anxiety and depression. More specifically, 74.2%, 83.87% and 70.97% of participants declaimed moderate to extremely severe stress, anxiety and depression, respectively. Since mental stress [18], dietary change [19] or circadian disruption [20] could all contribute to the exacerbation of skin concerns, the results of this current study investigated the comprehensive effect of fatigue rather than individual influence of the above factors.

Regarding self‐perceived fatigue‐induced skin issues, the top 5 concerns of participants were dullness, dark circles, increased fine lines, increased wrinkles, and acne. Interestingly, the clinical results via clinical grading and instrumental measurements were highly consistent with participants' self‐perception.

The perception of skin dullness could be influenced by colorimetric, optical, and skin surface microtopography parameters. As proposed, skin dullness would increase due to the decrease of skin redness and increase of skin yellowing [21, 22]. As reported by Matsubara, sleep shortage acted as a core factor in increasing facial yellowness and a delayed decrease of redness was observed after total sleep deprivation [23]. As shown in the current clinical study, both a* and b* values were significantly altered during workdays with a similar trend, which might jointly contribute to the sensation of dullness (Figures 5B and 4C).

In terms of skin aging‐related signs, increased fine lines around both periocular and cheek areas, intensified forehead wrinkles, and loss of skin elasticity were observed during workdays (Figures 4A–C and 5D–F). These observations suggested that fatigue could be an inducer of aging‐related signs. As was reported in early studies investigating the impact of a single day's work on skin aging, the facial signs including glabellar wrinkles, nasolabial fold, and crow's feet wrinkles among Caucasian subjects, as well as nasolabial, periorbital, and interocular wrinkles among the Asian population, were found aggravated after a working day [12, 13]. The results from facial photograph scoring in these studies were well accorded with and corroborated our instrument measuring results, confirming the prominent role of fatigue in accelerating skin aging.

Acne was also considered one of the most concerning skin issues by the participants. Porphyrins are metabolic products of Propionibacterium, contributing to the activation of the inflammatory response at perifollicular areas by upregulating IL‐8 and exerting cytotoxicity [24]. Thus far, it was believed that porphyrins could be closely linked to the risk of acne occurrence. In our study, porphyrin levels showed an increase versus the baseline on Sunday, which indicated the possible relation between fatigue and acne. As pointed out by Yang through a thorough review [25], lifestyle including diet, quality of sleep, and smoking, as well as psychological factors could all be related to acne. It was thus speculated that fatigue originated from an intensive work‐life pattern, which is a mix of negative lifestyle consequences, might also be closely related to acne.

As an inescapable stressor of modern life, fatigue impacts skin's health and appearance. This work is a pilot study analyzing one group with a highly intensive work‐life pattern, and some limitations must be considered. As the current design focused only on the 1 week change of facial traits, it could not yet determine whether the fatigue impact is periodic between weeks or it might be further aggravated in the long term. In addition, it remains unknown if participants with similar exhausting work‐life patterns, yet different levels of fatigue sensations, working conditions, or individual lifestyles, would exhibit similar changes in skin phenotypes. Therefore, an increase in sample size would provide us with more subgroups to identify such differences. Moreover, in this current study, only clinical measurements and questionnaires were performed, while it would bring more insights if biochemical markers can be detected in the future study.

In summary, throughout an entire workday‐rest day cycle in 1 week, we identified that the fatigue could be accumulated, as accompanied by worsened skin phenotypes. The limitations above call for further studies to investigate long‐term effects of fatigue, and integration of additional methodology and biochemical markers, to improve the specificity of the findings.

## 5 Conclusion

This study is a pioneering attempt to reveal the impact of fatigue on skin phenotype. Among highly exhausted subjects, it was found that the “996” intensive work‐life pattern increased the fatigue sensation during weekdays and aggravated aging signs such as fine lines and wrinkles, altered skin color, and increased risk of acne occurrence. These findings may provide insights on fatigued skin and help to optimize formulations specifically designed to address such concerns.

## Author Contributions

Wencai Jiang executed the clinical test. Xiaoyu Ma and Li Ma performed the data analysis. Li Ma, Yimei Tan, and Olivier Doucet designed the studies and interpreted the clinical results. Xiaoyu Ma and Li Ma wrote the manuscript. Pujos Muriel, Florante Ricarte, and Olivier Doucet reviewed the manuscript.

## Funding

This work was supported by Coty Inc.

## Ethics Statement

The study was performed in accordance with the ethical standards of the Helsinki Declaration. This clinical research was approved by the ethics committee of the Shanghai Skin & Disease Hospital [2024–19(妆)]. All recruited subjects provided written consent forms.

## Conflicts of Interest

Xiaoyu Ma, Li Ma, Pujos Murie, Florante Ricarte, and Doucet Olivier are employees of Coty Inc. Yimei Tan and Wencai Jiang from Shanghai Skin Disease Hospital executed the clinical study funded by Coty Inc.

## Supporting information


**Figure S1:** DASS‐21 distribution of participants.
**Table S1:** Chalder fatigue questionnaire.
**Table S2:** Lifestyle questionnaire.

## Data Availability

The data that support the findings of this study are available on request from the corresponding author. The data are not publicly available due to privacy or ethical restrictions.

## References

[1] F. Pega , B. Náfrádi , N. C. Momen , et al., “Global, Regional, and National Burdens of Ischemic Heart Disease and Stroke Attributable to Exposure to Long Working Hours for 194 Countries, 2000–2016: A Systematic Analysis From the WHO/ILO Joint Estimates of the Work‐Related Burden of Disease and Injury,” Environment International 154 (2021): 106595. 34011457 10.1016/j.envint.2021.106595 PMC8204267

[2] J. A. Caldwell , J. L. Caldwell , L. A. Thompson , and H. R. Lieberman , “Fatigue and Its Management in the Workplace,” Neuroscience and Biobehavioral Reviews 96 (2019): 272–289. 30391406 10.1016/j.neubiorev.2018.10.024

[3] A. M. Lock , D. L. Bonetti , and A. D. K. Campbell , “The Psychological and Physiological Health Effects of Fatigue,” Occupational Medicine (London) 68, no. 8 (2018): 502–511. 10.1093/occmed/kqy109 30445654

[4] I. M. Skoie , T. Ternowitz , G. Jonsson , K. Norheim , and R. Omdal , “Fatigue in Psoriasis: A Phenomenon to Be Explored,” British Journal of Dermatology 172, no. 5 (2015): 1196–1203. 25557165 10.1111/bjd.13647

[5] Y. Sawada , N. Saito‐Sasaki , E. Mashima , and M. Nakamura , “Daily Lifestyle and Inflammatory Skin Diseases,” International Journal of Molecular Sciences 22, no. 10 (2021): 5204. 34069063 10.3390/ijms22105204 PMC8156947

[6] Y. Chen and J. Lyga , “Brain‐Skin Connection: Stress, Inflammation and Skin Aging,” Inflammation & Allergy Drug Targets 13, no. 3 (2014): 177–190. 24853682 10.2174/1871528113666140522104422 PMC4082169

[7] C. M. Lee , R. E. B. Watson , and C. E. Kleyn , “The Impact of Perceived Stress on Skin Ageing,” Journal of the European Academy of Dermatology and Venereology 34, no. 1 (2020): 54–58. 31407395 10.1111/jdv.15865

[8] M. Pujos , C. Chamayou‐Robert , M. Parat , et al., “Impact of Chronic Moderate Psychological Stress on Skin Aging: Exploratory Clinical Study and Cellular Functioning,” Journal of Cosmetic Dermatology 24 (2024): e16634. 39506493 10.1111/jocd.16634 PMC11743297

[9] M. S. Matsui , E. Pelle , K. Dong , and N. Pernodet , “Biological Rhythms in the Skin,” International Journal of Molecular Sciences 17, no. 6 (2016): 801. 27231897 10.3390/ijms17060801 PMC4926335

[10] T. Yoshizaki , Y. Kimira , H. Mano , et al., “Association Between Skin Condition and Sleep Efficiency in Japanese Young Adults,” Journal of Nutritional Science and Vitaminology (Tokyo) 63, no. 1 (2017): 15–20. 10.3177/jnsv.63.15 28367921

[11] D. Léger , C. Gauriau , C. Etzi , et al., “‘You Look Sleepy…’ the Impact of Sleep Restriction on Skin Parameters and Facial Appearance of 24 Women,” Sleep Medicine 89 (2022): 97–103. 34971928 10.1016/j.sleep.2021.11.011

[12] F. Flament , J. Pierre , K. Delhommeau , and A. S. Adam , “How a Working Day‐Induced‐Tiredness May Alter Some Facial Signs in Differently‐Aged Caucasian Women,” International Journal of Cosmetic Science 39, no. 5 (2017): 467–475. 28295411 10.1111/ics.12398

[13] F. Flament , H. Qiu , A. Abric , and A. Charbonneau , “Assessing Changes in Some Facial Signs of Fatigue in Chinese Women, Induced by a Single Working Day,” International Journal of Cosmetic Science 41, no. 1 (2019): 21–27. 30488456 10.1111/ics.12506

[14] M. Cella and T. Chalder , “Measuring Fatigue in Clinical and Community Settings,” Journal of Psychosomatic Research 69, no. 1 (2010): 17–22. 20630259 10.1016/j.jpsychores.2009.10.007

[15] C. Sutherland , A. Smallwood , T. Wootten , and N. Redfern , “Fatigue and Its Impact on Performance and Health,” British Journal of Hospital Medicine (London, England) 84, no. 2 (2023): 1–8. 10.12968/hmed.2022.0548 36848155

[16] A. Descatha , G. Sembajwe , F. Pega , et al., “The Effect of Exposure to Long Working Hours on Stroke: A Systematic Review and Meta‐Analysis From the WHO/ILO Joint Estimates of the Work‐Related Burden of Disease and Injury,” Environment International 142 (2020): 105746. 32505015 10.1016/j.envint.2020.105746

[17] D. W. Johnston , J. L. Allan , D. J. H. Powell , et al., “Why Does Work Cause Fatigue? A Real‐Time Investigation of Fatigue, and Determinants of Fatigue in Nurses Working 12‐Hour Shifts,” Annals of Behavioral Medicine 53, no. 6 (2019): 551–562. 30124742 10.1093/abm/kay065

[18] M. Rinnerthaler , J. Bischof , M. K. Streubel , A. Trost , and K. Richter , “Oxidative Stress in Aging Human Skin,” Biomolecules 5, no. 2 (2015): 545–589. 25906193 10.3390/biom5020545 PMC4496685

[19] Y. S. Soliman , P. W. Hashim , A. S. Farberg , and G. Goldenberg , “The Role of Diet in Preventing Photoaging and Treating Common Skin Conditions,” Cutis 103, no. 3 (2019): 153–156. 31039233

[20] J. Duan , E. N. Greenberg , S. S. Karri , and B. Andersen , “The Circadian Clock and Diseases of the Skin,” FEBS Letters 595, no. 19 (2021): 2413–2436. 34535902 10.1002/1873-3468.14192 PMC8515909

[21] A. M. Nurani , K. Kikuchi , M. Iino , et al., “Development of a Method for Evaluating Skin Dullness: A Mathematical Model Explaining Dullness by the Color, Optical Properties, and Microtopography of the Skin,” Skin Research and Technology 29, no. 7 (2023): e13407. 37522508 10.1111/srt.13407 PMC10337531

[22] Y. Ogura , T. Kuwahara , M. Akiyama , et al., “Dermal Carbonyl Modification Is Related to the Yellowish Color Change of Photo‐Aged Japanese Facial Skin,” Journal of Dermatological Science 64, no. 1 (2011): 45–52. 21798719 10.1016/j.jdermsci.2011.06.015

[23] A. Matsubara , G. Deng , L. Gong , et al., “Sleep Deprivation Increases Facial Skin Yellowness,” Journal of Clinical Medicine 12, no. 2 (2023): 615. 36675544 10.3390/jcm12020615 PMC9861417

[24] C. Borelli , K. Merk , M. Schaller , et al., “In Vivo Porphyrin Production by *P. acnes* in Untreated Acne Patients and Its Modulation by Acne Treatment,” Acta Dermato‐Venereologica 86, no. 4 (2006): 316–319. 16874416 10.2340/00015555-0088

[25] J. Yang , H. Yang , A. Xu , and L. He , “A Review of Advancement on Influencing Factors of Acne: An Emphasis on Environment Characteristics,” Frontiers in Public Health 8 (2020): 450. 33042936 10.3389/fpubh.2020.00450 PMC7527424

