# Supplementary material for: Impact of Fatigue on the Skin: Pilot Study on “996” Work‐Life Pattern in China and the Influence of Their Lifestyle on the Skin
Source: J Cosmet Dermatol. 2026 Jan 21;25(1):e70679. doi: 10.1111/jocd.70679 (PMC12824052; doi:10.1111/jocd.70679)
Supplement: Supplementary file 1 — Figure S1: DASS‐21 distribution of participants. Table S1: Chalder fatigue questionnaire. Table S2: Lifestyle questionnaire. [file JOCD-25-e70679-s001.docx]

**Impact of fatigue on the skin: pilot study on “996” work-life pattern in China and the influence of their lifestyle on the skin**

**Table S1** **Chalder fatigue questionnaire^[1]^**

**Table S1** **Chalder fatigue questionnaire**

**chalder fatigue scale**

name: ______________________________ date: _______

We would like to know more about any problems you have had with feeling tired, weak or lacking in energy in the last month. Please answer ALL the questions by ticking the answer which applies to you most closely. If you have been feeling tired for a long while, then compare yourself to how you felt when you were last well. Please tick only one box per line.

|  | **less than usual** | **no more than usual** | **more than usual** | **much more than usual** |
| --- | --- | --- | --- | --- |
| do you have problems with tiredness? |  |  |  |  |
| do you need to rest more? |  |  |  |  |
| do you feel sleepy or drowsy? |  |  |  |  |
| do you have problems starting things? |  |  |  |  |
| do you lack energy? |  |  |  |  |
| do you have less  strength in your muscles? |  |  |  |  |
| do you feel weak? |  |  |  |  |
| do you have difficulties concentrating? |  |  |  |  |
| do you make slips of the  tongue when speaking? |  |  |  |  |
| do you find it more difficult to find the right word? |  |  |  |  |
|  | **better than usual** | **no worse than usual** | **worse than usual** | **much worse than usual** |
| how is your memory? |  |  |  |  |

Note: This scale can be scored “bimodally” with columns representing 0, 0, 1 & 1 and a range from 0 to 11 with a total of 4 or more qualifying for “caseness”.

**Table S2** **Lifestyle questionnaire**

Q14 is referred to DASS-21 questionnaire^[2]^ (The Depression Anxiety Stress Scales 21) , as a short form of original 42-item self-report measure of depression, anxiety, and stress (DASS).

**Table S2 Lifestyle questionnaire**

**Lifestyle questionnaire**

Q1.What is your skin type?（single choice）

| Oily | 1 |
| --- | --- |
| Dry | 2 |
| Oily to combination | 3 |
| Dry to combination | 4 |
| Neutral | 5 |

Q2.Sensitivity of your skin（single choice）

| Non-sensitive | 1 |
| --- | --- |
| Slightly-sensitive | 2 |
| Moderate-sensitive | 3 |
| Severe-sensitive | 4 |
| Don’t know/Not sure | 5 |

Q3.What’s your occupation（single choice）

| Heads or principals of national and social organizations, enterprises and institutions | 1 |
| --- | --- |
| Professionals & technicians | 2 |
| Clerical and related personnel | 3 |
| Social production service and consumer service personnel | 4 |
| Agriculture, forestry, animal husbandry, fishery production and auxiliary personnel | 5 |
| Manufacturing and related personnel | 6 |
| Military personnel | 7 |
| Other classifications | 8 |
| No job | 9 |

** According to the occupational classification system of the People's Republic of China (2022 edition)*

Q4. (Only for subjects selected Q3-8) What is your specific occupation? (Open question)

_________________________________

Q5. How long is your career? (single choice)

| Never | 1 |
| --- | --- |
| 1-5 years | 2 |
| 6-10 years | 3 |
| 11-15 years | 4 |
| Over 16 years | 5 |

Q6. How many children do you have? (single choice)

| 0 | 1 |
| --- | --- |
| 1 | 2 |
| 2 | 3 |
| more than 3 (including 3) | 4 |

Q7. How do you usually commute? (multiple choices)

| Walk | 1 |
| --- | --- |
| Bicycle or electric vehicle | 2 |
| Bus | 3 |
| Subway | 4 |
| Drive by oneself | 5 |
| Taxi/sharing taxi with others | 6 |
| Home base | 7 |
| Else：_____________ | 8 |

Q7a. (Only for subjects selected Q7-8) What is your specific occupation? (Open question)

_____________________________________________________________________

Q8. What bothers you most under intense, high-intensity work? (multiple choices)

| Sleep issue | 1 |
| --- | --- |
| Diet issue | 2 |
| Physical issue | 3 |
| Mental issue | 4 |
| Social issue | 5 |
| Else : _____________ | 6 |

Q8a. (Only for subjects selected Q8-1) What are the main sleep problems caused by intense work? (multiple choices)

| Irregular sleep | 1 |
| --- | --- |
| Difficult to fall asleep | 2 |
| Dreaminess | 3 |
| Light sleep | 4 |
| Insomnia | 5 |
| Else: _____________ | 6 |

Q8b.（Only for subjects selected Q8-2）What are the main dietary problems caused by intense work? (multiple choices)

| No appetite | 1 |
| --- | --- |
| Overeating | 2 |
| Unbalanced nutrition | 3 |
| Prefer high calories | 4 |
| Heavy flavor | 5 |
| Else: _____________ | 6 |

Q8c.（Only for subjects selected Q8-3）What are the main physical problems caused by intense work? (multiple choices)

| Hair loss | 1 |
| --- | --- |
| Obesity | 2 |
| Skin issue | 3 |
| Endocrine disorders | 4 |
| High blood pressure, lipid and blood sugar | 5 |
| Immunity | 6 |
| Gastrointestinal discomfort | 7 |
| Else: _____________ | 8 |

Q8d.（Only for subjects selected Q8-4）What are the main mental problems caused by intense work? (multiple choices)

| Loss of memory | 1 |
| --- | --- |
| Anxiety | 2 |
| Irascibility | 3 |
| Low spirit | 4 |
| Instable emotion | 5 |
| Else:_____________ | 6 |

Q8e.（Only for subjects selected Q8-5）What are the main social problems caused by intense work? (multiple choices)

| Lack friend reunion | 1 |
| --- | --- |
| Lack family companionship | 2 |
| Else:_____________ | 3 |

Q9.（Only for subjects selected Q8c-3）What are the main skin problems caused by intense work? (multiple choices and no more than 5 selections)

| Increase of fine line | 1 |
| --- | --- |
| Increase of wrinkle | 2 |
| Acne | 3 |
| Dark circle | 4 |
| Puffy eyes | 5 |
| Puffy face | 6 |
| Dullness | 7 |
| Yellowness | 8 |
| Skin laxity | 9 |
| Redness | 10 |
| Unevenness | 11 |
| Roughness | 12 |
| Sensitivity(redness, sting, itch) | 13 |
| Dryness and desquamation | 14 |
| Oily | 15 |
| Coarse pores | 16 |
| Spot | 17 |
| Else: _____________ | 18 |

Q10.（Only for subjects selected Q8c-3）What are the main approaches you seek for to improve the skin problems caused by intense work? (multiple choices and no more than 5 selections)

| Skincare products | 1 |
| --- | --- |
| Makeups | 2 |
| Facial mask | 3 |
| Beauty massage | 4 |
| Medical procedure | 5 |
| Home-based device | 6 |
| Diet | 7 |
| Exercise | 8 |
| Supplements | 9 |
| Chinese medicine | 10 |
| Life style adjustment | 11 |
| Else: _____________ | 12 |

Q11.（Only for subjects selected Q8c-3 and Q16-1）What skincare products do you use to improve the skin problems caused by intense work? (Multiple choices and no more than 5 selections)

| Toner | 1 |
| --- | --- |
| Toning lotion | 2 |
| Eye serum | 3 |
| Eye mask | 4 |
| Essence oil | 5 |
| Essence lotion | 6 |
| Serum | 7 |
| Lotion | 8 |
| Cream | 9 |
| Mask | 10 |
| Eye cream | 11 |
| Else: _____________ | 12 |

Q12.（Only for subjects selected Q8c-3 and Q16-1）What are the most expected efficacy for skincare products to improve the skin problems caused by intense work?（Multiple choices and no more than 5 selections）

| Reduce fine line & wrinkle | 1 |
| --- | --- |
| Improve acne | 2 |
| Improve dark circle | 3 |
| Reduce puffiness | 4 |
| Reduce yellowness & increase brightness | 5 |
| Skin whitening | 6 |
| Tighten skin | 7 |
| Reduce redness | 8 |
| Promote skin color evenness | 9 |
| Promote smoothness | 10 |
| Maintain stability | 11 |
| Moisturizing | 12 |
| Reduce sebum | 13 |
| Refine skin pores | 14 |
| Improve dark spot | 15 |
| Else: _____________ | 16 |

Q13. How much time do you spend on the following activities? (Please answer based on your situation in the last three months)（fill in the blanks）

|  |  | Workday(Mon-Fri) | Weekend(Sat & Sun) |
| --- | --- | --- | --- |
| 13-1 | Work | ___ hr/day | ___ hr/day |
| 13-2 | Exercise/fitness | ___ hr/day | ___ hr/day |
| 13-3 | Entertainment (games, TV, etc.) | ___ hr/day | ___ hr/day |
| 13-4 | Social media | ___ hr/day | ___ hr/day |
| 13-5 | Sleep | ___ hr/day | ___ hr/day |
| 13-6 | Outdoor time (exposure to the sun) | ___ hr/day | ___ hr/day |
| 13-7 | Cook | ___ hr/day | ___ hr/day |
| 13-8 | Use computer, cell phone, tablet computer, etc | ___ hr/day | ___ hr/day |
| 13-9 | Commute | ___ hr/day | ___ hr/day |
| 13-10 | Stay with family/children | ___ hr/day | ___ hr/day |

Q14. Please read each sentence carefully and circle the number to the right to select the best answer based on your experience in the past three months. There are no right or wrong answers. Please don't spend too much time on one sentence (DASS-21)

|  |  | Never | Sometimes | Often | Always |
| --- | --- | --- | --- | --- | --- |
| 14-1 | I find it hard to calm myself down | 0 | 1 | 2 | 3 |
| 14-2 | I was aware of dryness of my mouth | 0 | 1 | 2 | 3 |
| 14-3 | I couldn’t seem to experience any positive feeling at all. | 0 | 1 | 2 | 3 |
| 14-4 | I experienced breathing  difficulty(Not in exercise) | 0 | 1 | 2 | 3 |
| 14-5 | I find it difficult to work up the initiative to do things | 0 | 1 | 2 | 3 |
| 14-6 | I tend to over-react to situations | 0 | 1 | 2 | 3 |
| 14-7 | I experienced trembling (e.g., in the hands) | 0 | 1 | 2 | 3 |
| 14-8 | I feel that I was using a lot of nervous energy | 0 | 1 | 2 | 3 |
| 14-9 | I was worried about situations in which I  might panic and make a fool of myself | 0 | 1 | 2 | 3 |
| 14-10 | I felt that I had nothing to look forward to | 0 | 1 | 2 | 3 |
| 14-11 | I find myself getting agitated | 0 | 1 | 2 | 3 |
| 14-12 | I find it is difficult to relax | 0 | 1 | 2 | 3 |
| 14-13 | I felt down-hearted and blue | 0 | 1 | 2 | 3 |
| 14-14 | I was unable to become enthusiastic about anything | 0 | 1 | 2 | 3 |
| 14-15 | I felt I was close to panic | 0 | 1 | 2 | 3 |
| 14-16 | I was intolerant of anything that kept me  from getting on with what I was doing | 0 | 1 | 2 | 3 |
| 14-17 | I felt I wasn’t worth much as a person | 0 | 1 | 2 | 3 |
| 14-18 | I feel that I was rather touchy | 0 | 1 | 2 | 3 |
| 14-19 | I was aware of the action of my heart in the absence of physical exertion | 0 | 1 | 2 | 3 |
| 14-20 | I feel scared without any good reason | 0 | 1 | 2 | 3 |
| 14-21 | I feel that life is meaningless | 0 | 1 | 2 | 3 |

The score of Q14 DASS-21 questionnaire will be calculated. Option number of 14-1, 6, 8, 11, 12, 14, 18 scores will need to be summed and multiplied by 2 to calculate the final score of Stress level. Option number of 14-2, 4, 7, 9, 15, 19, 20 scores will need to be summed and multiplied by 2 to calculate the final score of Anxiety level. Option number of 14-3, 5, 8, 10, 13, 16, 17, 21 scores will need to be summed and multiplied by 2 to calculate the final score of Depression level. The Stress, Anxiety and Depression level of respective scores is shown as below table.

| Level | Stress | Anxiety | Depression |
| --- | --- | --- | --- |
| Normal | 0-14 | 0-7 | 0-9 |
| Mild | 15-18 | 8-9 | 10-13 |
| Moderate | 19-25 | 10-14 | 14-20 |
| Severe | 26-33 | 15-19 | 21-27 |
| Extremely Severe | 34+ | 20+ | 28+ |

**Figure S1** **DASS-21 distribution of participants**

Figure S1: DASS-21 distribution of participants

**References:**

1. Cella M, Chalder T. Measuring fatigue in clinical and community settings. J Psychosom Res. 2010;69(1):17-22.
2. Henry JD, Crawford JR. The short-form version of the Depression Anxiety Stress Scales (DASS-21): construct validity and normative data in a large non-clinical sample. Br J Clin Psychol. 2005;44(Pt 2):227-239.
